# 

**Published:** 1909-05

**Authors:** 


**CONTENTS—MAY, 1909.—VOL. XXIV, NO. 11.**

ORIGINAL ARTICLES—

The Anti-Tuberculosis Crusade and Phthisiophobia. By Theo. Y. Hull, M. D., San Antonio, Texas ........... 453

The Pathology of Fear. By W. T. Marrs, M. D., Peoria, Ill. ........................................ 461

EDITORIAL DEPARTMENT—

The Annual Meeting of the Texas State Medical Association ........................................ 463

The Lifting of the Veil ........................... 463

Dr. Fly's Letter, and What Has Come of It,—So Far.... 469

CORRESPONDENCE ........................................ 474

ABSTRACTS AND SELECTIONS ........................... 479

PUBLISHER'S DEPARTMENT ........................... 491

